# High-resolution genome-wide allelotype analysis identifies loss of chromosome 14q as a recurrent genetic alteration in astrocytic tumours

**DOI:** 10.1038/sj.bjc.6600430

**Published:** 2002-07-02

**Authors:** J Hu, JC-s Pang, CY-k Tong, B Lau, X-l Yin, W-S Poon, C-C Jiang, L-F Zhou, H-K Ng

**Affiliations:** Department of Neurosurgery, Hua Shan Hospital, Shanghai Medical University, Shanghai, China; Department of Anatomical & Cellular Pathology, Prince of Wales Hospital, The Chinese University of Hong Kong, Hong Kong, China; Neurosurgical Unit, Department of Surgery, Prince of Wales Hospital, The Chinese University of Hong Kong, Hong Kong, China

**Keywords:** fibrillary astrocytoma, glioblastoma multiforme, allelotyping, loss of heterozygosity

## Abstract

Diffusely infiltrative astrocytic tumours are the most common neoplasms in the human brain. To localise putative tumour suppressor loci that are involved in low-grade astrocytomas, we performed high-resolution genome-wide allelotype analysis on 17 fibrillary astrocytomas. Non-random allelic losses were identified on chromosomal arms 10p (29%), 10q (29%), 14q (35%), 17p (53%), and 19q (29%), with their respective common regions of deletions delineated at 10p14-15.1, 10q25.1-qter, 14q212.2-qer, 17p11.2-pter and 19q12-13.4. These results suggest that alterations of these chromosomal regions play important roles in the development of astrocytoma. We also allelotyped 21 *de novo* glioblastoma multiforme with an aim to unveil genetic changes that are common to both types of astrocytic tumours. Non-random allelic losses were identified on 9p (67%), 10p (62%), 10q (76%), 13q (60%), 14q (50%), and 17p (65%). Allelic losses of 10p, 10q, 14q and 17p were common genetic alterations detectable in both fibrillary astrocytomas and glioblastoma multiforme. In addition, two common regions of deletions on chromosome 14 were mapped to 14q22.3-32.1 and 14q32.1-qter, suggesting the presence of two putative tumour suppressor genes. In conclusion, our comprehensive allelotype analysis has unveiled several critical tumour suppressor loci that are involved in the development of fibrillary astrocytomas and glioblastoma multiforme. Although these two types of brain tumours are believed to evolve from different genetic pathways, they do share some common genetic changes. Our results indicate that deletions of chromosome 14q is a recurrent genetic event in the development of astrocytoma and highlight the subchromosomal regions on this chromosome that are likely to contain putative tumour suppressor genes involved in the oncogenesis of astrocytic tumours.

*British Journal of Cancer* (2002) **87**, 218–224. doi:10.1038/sj.bjc.6600430 www.bjcancer.com

© 2002 Cancer Research UK

## 

Diffusely infiltrating astrocytomas are the most frequent intracranial neoplasms and account for more than 60% of all primary brain tumours. According to the World Health Organization (WHO) classification, diffuse astrocytomas are graded into fibrillary astrocytoma (grade II), anaplastic astrocytoma (grade III) and glioblastoma multiforme (GBM; grade IV) (Kleihues *et al*, 2000). The fibrillary astrocytomas tumours have the inherent tendency for malignant progression to higher grade, with GBM as the most malignant phenotypic endpoint. Such glioblastomas are termed secondary GBM, in contrast to those *de novo* GBM that arise without prior known low-grade astrocytic tumours.

Molecular genetic studies on astrocytic tumours have been performed extensively to investigate the common events involved. In low-grade astrocytic tumours, chromosomal regions with frequent loss have been identified on 6q, 10p, 13q, 17p and 22q (Tsuzuki *et al*, 1996; Ino *et al*, 1999; Miyakawa *et al*, 2000). Mutation of the TP53 gene, which is located on 17p, is the most common genetic alteration detected in >60% tumours (von Deimling *et al*, 1992a). Gains of chromosomal arms 7q and 8q are also detected in a subset of low-grade astrocytic tumours (Schrock *et al*, 1996; Nishizaki *et al*, 1998). Moreover, low-grade astrocytic tumours often recur with concomitant malignant progression to anaplastic astrocytomas or glioblastomas by acquiring additional genetic changes. These alterations include 19q loss, RB1 alteration, 10q loss, PTEN/MMAC1 mutations, loss of DCC expression, and overexpression of PDGFR-α (Hermanson *et al*, 1992; von Deimling *et al*, 1992b; Karlbom *et al*, 1993; Henson *et al*, 1994; Li *et al*, 1997; Reyes-Mugica *et al*, 1997; Steck *et al*, 1997). On the other hand, *de novo* GBM is characterised by a distinct set of genetic changes that involve gene amplification and overexpression of EGFR and MDM2, CDKN2A deletion, 10p and 10q loss, and RB1 alteration (Libermann *et al*, 1985; Karlbom *et al*, 1993; Reifenberger *et al*, 1993; Schmidt *et al*, 1994; Ueki *et al*, 1996).

Molecular genetic studies on low-grade astrocytic tumours have been limited and these studies focused mostly on specific chromosomes and genes that are commonly affected in other tumour types. It still remains unknown whether other autosomes are involved in the development of astrocytomas. In this study, we performed a comprehensive genome-wide allelotype analysis on 17 low-grade fibrillary astrocytomas, using 382 microsatellite markers that cover the 22 autosomes, with an aim to detect critical tumour suppressor loci that are involved in astrocytoma formation. We also allelotyped 21 *de novo* GBM to identify genetic events that are common to both types of astrocytic tumours.

## MATERIALS AND METHODS

### Specimens

Seventeen fibrillary astrocytomas and 21 *de novo* GBM were collected from hospitals in Hong Kong and China. Tumours obtained from surgery were immediately stored at −80°C until use. All tumours were diagnosed according to the recent WHO criteria (Kleihues *et al*, 2000). Each tumour was histologically confirmed to have neoplastic cell content greater than 80%. The corresponding peripheral blood of each patient was also obtained as constitutional control. The mean age of patients with fibrillary astroytomas was 34±7.3 years and the male/female ratio was 1.1, whereas patients with GBM tended to be older with a mean age of 45.2±14.5 years and their male/female ratio was 0.9. None of the cases were recurrences.

### DNA extraction

About 20–30 pieces of 10 μm thick frozen sections of each tumour were cut for DNA extraction. High molecular weight genomic DNA from both blood and tumour was purified using conventional proteinase K digestion and phenol-chloroform extraction.

### Allelotype analysis

A high-resolution genome-wide allelotype analysis was performed according to reported protocols (Tong *et al*, 2001). Briefly, 382 microsatellite loci derived from 22 autosomes were examined for allelic imbalances. The average interval of these loci is about 10 cM. The polymorphic microsatellite markers were obtained from the ABI Prism Linkage Mapping Set V.2 (Applied Biosystems, CA, USA) and were originally selected from the Généthon human linkage map. The set consists of primer pairs end-labelled with either one of three fluorochromes: FAM, HEX, or NED. PCR was performed in a final volume of 7.5 μl containing two primer pairs (2.5 pmoles of each primer), 60 ng of DNA, 10 mM Tris-HCl (pH 8.3), 50 mM KCl, 0.2 mM deoxyribonucleoside triphosphates, 2.5 mM MgCl_2_ and 0.6 unit of AmpliTaq Gold DNA polymerase (Applied Biosystems). To facilitate high throughput of samples, all liquid handling and thermal cycling were carried out in an ABI Prism 877 robotic workstation (Applied Biosystems). PCR was started, according to manufacturer's recommendation, with 95°C for 15 min, followed by 10 cycles composed of 94°C for 15 s, 55°C for 15 s and 72°C for 30 s, and another 22 cycles composed of 89°C for 15 s, 55°C for 15 s and 72°C for 30 s. Amplified PCR products of multiple loci were pooled and electrophoresed in denaturing 5% polyacrylamide gels on an ABI Prism 377 automated DNA sequencer (Applied Biosystems). The data collected were analysed using GeneScan Analysis software version 3.1 (Applied Biosystems). Allelic imbalance was defined by calculating the allelic ratio (AR) of both normal (N) and tumour (T) DNA, where AR was the ratio of peak height of the longer allele (N2 or T2) to that of the shorter allele (N1 or T1), i.e., AR=(N2/N1)/(T2/T1). Allelic imbalance was indicated when the ratio was greater than 1.5 or smaller than 0.5, representing loss of longer or shorter allele respectively.

## RESULTS

**Figure 1:**
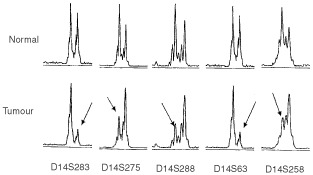
Representative results of allelotype analysis. Allelic patterns of five polymorphic loci on chromosomal arm 14q examined in a fibrillary astrocytoma (case 21) are shown. Allelic loss is indicated by arrow.

To localise critical tumour suppressor loci involved in the tumorigenesis of astrocytoma, we performed a comprehensive genome-wide allelotype analysis on 17 fibrillary astrocytomas and 21 GBM. All 22 autosomes were examined for allelic imbalances. An average of 70% informative loci/case was detected in our series. Allelic imbalances were seen in all 39 autosomal arms. Representative results of allelic imbalances at selected microsatellite loci are illustrated in Figure 1

.

### Allelotype of fibrillary astrocytomas

**Figure 2:**
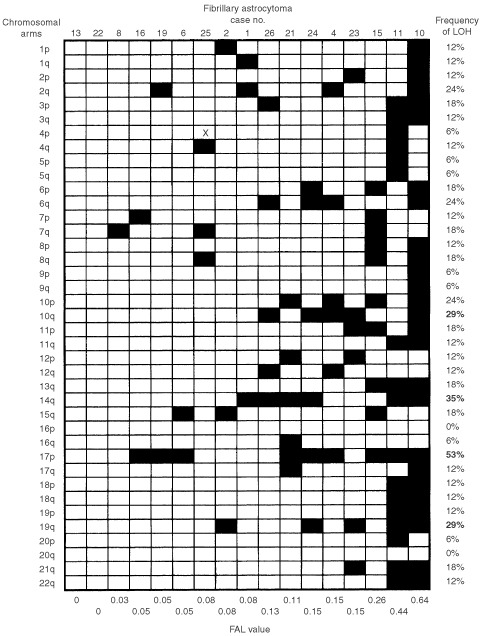
Summary of allelic imbalances detected in 17 fibrillary astrocytoma. Case number is indicated on top and fractional allelic loss (FAL) value is shown at bottom. Frequency of LOH is indicated on right, with bold number representing non-random allelic imbalance frequency above the baseline level (25%). Filled box represents allelic imbalance detected in specified chromosomal arm and open box indicate chromosomal arm with no detectable allelic imbalance. X, not done.

The frequency of allelic imbalances for individual chromosomal arm in the low-grade astrocytoma series varied from 0% (12q, 16p, 20q and 22q) to 53% (17p) (Figure 2

). The mean percentage of allelic imbalances was 15±10%. In the present study, 25% (mean percentage+one standard deviation) was chosen to be a significant percentage of allelic imbalances. This represents the 95% confidence upper limit for the overall rate for random chromosome loss or imbalance in tumours. Non-random allelic imbalances above the baseline (25%) were identified on chromosomal arms 10p (29%), 10q (29%), 14q (35%), 17p (53%), and 19q (29%). Comparative genomic hybridisation (CGH) analysis was also performed on three tumours, in which sufficient DNA was available. These tumours were confirmed to have deletion regions identified by allelotyping (data not shown).

**Figure 4:**
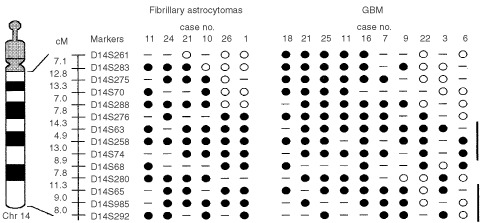
Delineation of common region of deletion on chromosomal arm 14q in astrocytic tumors. Fourteen polymorphic loci, with their respective genetic intervals in centiMorgan (cM), were examined for allelic loss. Two common regions of deletion (thick bars) are identified: 14q22.3-32.1 and 14q32.1-qter. Filled circle represents loss of heterozygosity and open circle denotes retention of heterozygosity. Dash line indicates homozygosity. The candidate tumour suppressor gene, MLH3, is located between markers D14S258 and D14S74 on the genetic map.

We have also delineated the common regions of deletion (CRDs) on chromosomes that are frequently lost in fibrillary astrocytomas. Two CRDs were mapped on chromosome 10, one was located to a region of 5.2 cM on 10p14-15.1 (between D10S591 and D10S189) and the other was mapped to a region of 42.4 cM between 10q25.1 (marker D10S597) and the telomere of long arm. The LOH frequency at this region was 29% (five of 17 informative cases). The CRD on chromosome 14q was localized to a region of 85 cM between 14q21.2 (D14S288) and the 14q telomere (Figure 4

). The LOH frequency at this region was 35% (six of 17 informative cases). On chromosome 17, one CRD was defined within 17p11.2 (D17S799) and the 17p telomere, with a LOH frequency of 53% (nine of 17 informative cases). Another CRD of 51.2 cM was delineated on chromosome 19q12-13.4, between markers D19S414 and D19S210. The LOH frequency at this region was 29% (five of 17 informative cases). In addition, chromosomal arms with frequencies of allelic imbalances higher than the mean percentage of LOH were identified on 2q (24%), 3p (18%), 6p (18%), 6q (24%), 7q (18%), 8q (18%), 10p (24%), 11p (18%), 13q (18%), 15q (18%) and 21q (18%).

### Allelotype of GBM

**Figure 3:**
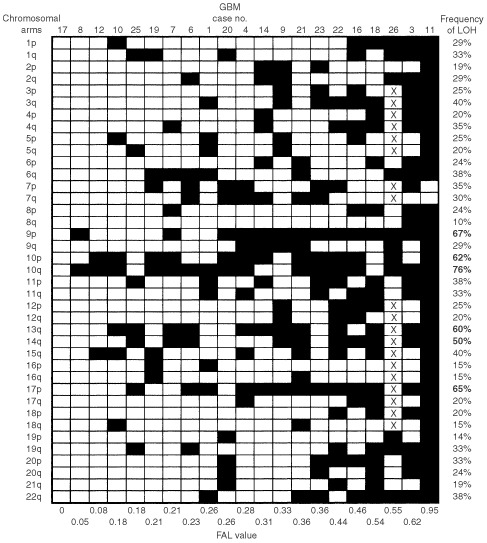
Summary of allelic imbalances detected in 21 GBM. Case number is indicated on top and fractional allelic loss (FAL) value is shown at bottom. Frequency of LOH is indicated on right, with bold number representing non-random allelic imbalance frequency above the baseline level (47.9%). Filled box represents allelic imbalance detected in specified chromosomal arm and open box indicate chromosomal arm with no detectable allelic imbalance. X, not done.

The frequency of allelic imbalances for individual chromosomal arm in the GBM series varied from 10% (8q) to 76% (10q) (Figure 3

). The mean percentage of allelic imbalance was 32±15.9%, with 47.9% (mean percentage+one standard deviation) chosen to be a significant percentage of allelic imbalances. Non-random allelic imbalances were detected on chromosomes 9p (67%), 10p (62%), 10q (76%), 13q (60%), 14q (50%) and 17p (65%).

The CRD of 11.1 cM on chromosome 9 was mapped to 9p21-23, flanked by markers D9S286 and D9S285. The LOH frequency at this region was 62%. Three CDRs were observed on chromosome 10, at 10p15-pter (16.5 cM between D10S189 and D10S249), 10q23.3-25.1 (9.9 cM flanked by D10S185 and D10S597), and 10q25.3-26.3 (30.6 cM between D10S1693 and D10S1651). The LOH frequencies at these deletion regions were 58% (11 of 19 informative cases), 77% (13 out of 17) and 58% (11 out of 19), respectively. On chromosome 13, the CDR lied in a region of 50.7 cM, between markers D13S217 and D13S170. The frequency of LOH at this 13q13.2-31 deletion region was 50% (10 out of 20). Two CRDs were located on chromosome 14: one was mapped to 14q22.3-32.1 (48.9 cM between D14S276 and D14S280) and the other was localised between 14q32.1 and 14q telomere (28.3 cM between D14S280 and pter), with LOH frequency of 50% (10 out of 20) and 44% (eight out of 18), respectively (Figure 4). Another CRD was observed between 17p12 (D17S1852) and 17p telomere (23 cM). This deletion region was detected in 60% (12 out of 20) of informative cases.

CGH analysis was performed on nine GBM and confirmed the deletion regions identified by allelotyping. Chromosomal regions that show gain of genetic material had been analysed by array-based CGH, in which 58 oncogenes/amplicons were investigated, and were reported elsewhere (Hui *et al*, 2001).

### Fractional allelic loss

The fractional allelic loss (FAL) for each tumour was also determined (Figures 2 and 3). FAL is defined as the fraction of the total number of informative chromosomal arms that displays allelic loss (Vogelstein *et al*, 1989). In the present study, the mean FAL values for fibrillary astrocytomas and GBM were 0.15 (range, 0–0.67) and 0.33 (range, 0–0.95), respectively. This result indicates that additional genetic alterations were accumulated in GBM. There was no association between the FAL values and the age/sex of the patients. By using Mann–Whitney *U*-test, high FAL value was found to be significantly associated with chromosomes 10q (*P*=0.05), 14q (*P*=0.01) and 19q (*P*=0.01) in fibrillary astrocytomas, and 9p (*P*<0.01), 14q (*P*=0.02) and 17p (*P*<0.01) in GBM. Moreover, microsatellite instability was rarely found in our series.

## DISCUSSION

In this study, we performed a comprehensive genome-wide allelotype analysis on a series of 17 low-grade astrocytomas and 21 *de novo* GBM with an aim to localise putative tumour suppressor loci involved in astrocytic tumours. Non-random chromosome losses were identified on 10p, 10q, 14q, 17p and 19q in fibrillary astrocytomas, and on 9p, 10p, 10q, 13q, 14q and 17p in GBM (Table 1

**Table 1:**
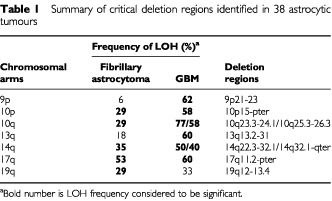
Summary of critical deletion regions identified in 38 astrocytic tumours

). These results strongly indicate that alterations of these chromosomes play critical roles in the genesis of astrocytoma.

Mutation of the TP53 gene and/or allelic loss of 17p is the most prominent genetic alteration detected in >60% of fibrillary astrocytomas (von Deimling *et al*, 1992a). Study on malignant progression of low-grade astrocytomas to high-grade tumours revealed that the frequencies of TP53 mutation were comparable in both groups of tumours, suggesting that TP53 mutation is an early event in the evolution of diffuse astrocytoma (Watanabe *et al*, 1997). Our allelotype analysis identifies a CRD between 17p11.2 and 17p telomere, which encompasses the TP53 locus, as the major genetic alteration in the low-grade astrocytomas.

In the present study, allelic loss of 14q is the second most common genetic alteration detected in fibrillary astrocytomas. Since chromosome 14 has never been a target under investigation for genetic changes in low-grade astrocytomas, we therefore reviewed genetic results from three genome-wide studies covering a total of 40 fibrillary astrocytomas (Schrock *et al*, 1996; Sallinen *et al*, 1997; von Deimling *et al*, 2000). Sallinen *et al* (1997) detected chromosome loss of 14q in 18% tumours by CGH, whereas Schrock *et al* (1996) (with CGH) and von Deimling *et al* (2000) (with allelotype analysis) detected no alteration in chromosome 14q. It should be noted that only three polymorphic markers on chromosome 14q were examined in the latter allelotyping study. Our allelotype analysis investigated 14 polymorphic loci on the long arm of chromosome 14 and detected 35% of tumours with 14q loss. Moreover, we were able to delineate a CRD between 14q21.2 and 14q telomere. Although low-grade astrocytomas and *de novo* GBM are regarded as two groups of brain tumours with distinct genetic changes, we did observe 14q loss in 50% of GBM in our series. In addition, two CRDs were localised on chromosome 14 in GBM, one mapped to 14q22.3-32.1 and the other defined within 14q32.1 and 14q telomere (Figure 4). The presence of two CRDs suggests that there are two tumour suppressor genes located on chromosome 14q. It seems that chromosome 14q loss is a common event for both low-grade astrocytomas and *de novo* GBM. Further study is needed to refine the deletion regions on chromosome 14q for positional cloning of the tumour suppressor genes involved in astrocytomas.

Frequent deletion of 14q has also been detected in a variety of tumours such as meningioma, neuroblastoma, breast and ovarian cancer (Suzuki *et al*, 1989; Bandera *et al*, 1997; Tse *et al*, 1997; O'Connell *et al*, 1999). Deletion mapping has identified at least three tumour suppressor loci on 14q that are important for cancer development. These regions are mapped to 14q11-13, 14q22-24 and 14q32 (Bandera *et al*, 1997; Tse *et al*, 1997; O'Connell *et al*, 1999). The latter two regions overlap with the deletion regions delineated in this study. Thus, deletion at 14q is a common feature in different tumour types including astrocytic tumours. A candidate tumour suppressor gene, MLH3, has been identified on 14q24.3. MLH3 is a mismatch-repair gene and mutation of this gene is associated with microsatellite instability in colorectal tumours (Lipkin *et al*, 2001). Other mismatch-repair genes such as MLH1 and MSH2 have been found to be mutated in a subset of malignant gliomas (Leung *et al*, 1998). Whether genetic alteration of MLH3 plays a role in astrocytoma formation remains to be determined.

Allelic loss of chromosome 19q was detected in 29% and 33% of fibrillary astrocytomas and GBM, respectively, in our allelotype analysis. These results are consistent with previous allelotyping study and suggest the presence of a tumour suppressor gene on 19q. Chromosome 19q loss is the only known genetic alteration that is shared by the three major glioma subtypes, astrocytomas, oligodendrogliomas and oligoastrocytomas (von Deimling *et al*, 1992b). The deletion breakpoint on 19q has been mapped to the segment 19q13.3. Recent finer deletion mapping have identified two overlapping regions, flanked by markers D19S241 and STD, with high frequency of allelic loss (Rosenberg *et al*, 1996; Smith *et al*, 2000a). A partial transcript map has been constructed for this deletion region (Smith *et al*, 2000b) and it remains to be determined if any of these genes is the tumour suppressor for gliomas.

Deletions of chromosome 10 are among the most frequent genetic alterations in astrocytomas, particularly in the high-grade variants. Several deletion regions have been identified on both arms of chromosome 10 and these regions have been mapped to 10p15, 10p14 and 10q25 (Rasheed *et al*, 1995; Voesten *et al*, 1997; Ichimura *et al*, 1998; Kon *et al*, 1998). The presence of tumour suppressor genes on these deletion regions is supported by chromosome transfer studies (Pershouse *et al*, 1993; Kon *et al*, 1998). Two putative tumour suppressor genes, namely PTEN/MMAC1 and DMBT1, have been identified on chromosome 10q23.3 and 10q25, respectively (Li *et al*, 1997; Mollenhauer *et al*, 1997; Steck *et al*, 1997). In this study, we detected chromosome 10 loss in seven (41%) fibrillary astrocytomas and 18 (86%) GBM, supporting the notion that chromosome 10 plays an important role in astrocytoma formation. Recent study also suggests that low-grade astrocytomas with chromosome 10 loss are likely to undergo malignant progression (Ichimura *et al*, 1998). Whether the fibrillary astroytomas examined in this study behave similarly requires further follow-up evaluation.

In the GBM series, we detected non-random chromosome losses on 9p13.3-21.3 (58%), 10p15-pter (77%), 10q23.3-25.1 (58%), 13q13.2-31 (50%), 14q22.3-32.1 (50%) and 17p12-pter (60%). We compared our results with the published allelotyping data (von Deimling *et al*, 2000) and found that the data, except for LOH 17q, from both studies were largely comparable. The frequency of 17q loss in *de novo* GBM is the highest frequency reported to date and this is consistent with another series of GBM that we examined in previous study (Cheng *et al*, 1999). The putative tumour suppressor genes on some of these chromosomes have been identified and their associated genetic pathways are elucidated. For instance, CDKN2A (on 9p21) and RB1 (on 13q14.2) are involved in the cell cycle control; p14^ARF^ (on 9p21) negatively regulates TP53 (on 17p13.1) function; and PTEN/MMAC1 (on 10q23.3) is involved in the PI3K/AKT pathway. These pathways are believed to play important roles in the development and progression of astrocytoma. Recently, Pieper's group using a functional approach demonstrated that normal astrocytes could be transformed to astrocytoma by interfering with the telomerase, RAS, CDKN2A/RB1 and TP53 pathways (Sonoda *et al*, 2001a). They further showed that additional disturbance of the PI3K/AKT pathway elicited a glioblastoma phenotype in the *in vitro* transformed astrocytoma (Sonoda *et al*, 2001b). Thus, conversion of normal astrocytes to glioblastoma requires disruptions of at least five genetic pathways. The roles of other tumour suppressor loci on 10p and 14q in astrocytoma formation remain to be elucidated.

In conclusion, our comprehensive allelotype analysis have unveiled several critical tumour suppressor loci that are involved in the development of fibrillary astrocytomas and GBM. Although these two types of brain tumours are believed to evolve from different genetic pathways, they do share some common genetic changes. Our results indicate that deletions of chromosome 14q is a recurrent genetic event in the development of astrocytoma and highlight the subchromosomal regions on this chromosome that are likely to contain putative tumour suppressor genes involved in the oncogenesis of astrocytic tumours.

## References

[bib1] BanderaCATakahashiHBehbakhtKLiuPCLiVolsiVABenjaminIMorganMAKingSARubinSCBoydJ1997 Deletion mapping of two potential chromosome 14 tumor suppressor gene loci in ovarian carcinoma Cancer Res 57 513515 9012483

[bib2] ChengYNgHKDingMZhangSFPangJCLoKW1999 Molecular analysis of microdissected *de novo* glioblastomas and paired astrocytic tumors J Neuropathol Exp Neurol 58 120128 10029095 10.1097/00005072-199902000-00002

[bib3] HensonJWSchnitkerBLCorreaKMvon DeimlingAFassbenderFXuHJBenedictWFYandellDWLouisDN1994 The retinoblastoma gene is involved in malignant progression of astrocytomas Ann Neurol 36 714721 7979217 10.1002/ana.410360505

[bib4] HermansonMFunaKHartmanMClaesson-WelshLHeldinCHWestermarkBNisterM1992 Platelet-derived growth factor and its receptors in human glioma tissue: expression of messenger RNA and protein suggests the presence of autocrine and paracrine loops Cancer Res 52 32133219 1317261

[bib5] HuiABLoKWYinXLPoonWSNgHK2001 Detection of multiple gene amplifications in glioblastoma multiforme using array-based comparative genomic hybridization Lab Invest 81 717723 11351043 10.1038/labinvest.3780280

[bib6] IchimuraKSchmidtEEMiyakawaAGoikeHMCollinsVP1998 Distinct patterns of deletion on 10p and 10q suggest involvement of multiple tumor suppressor genes in the development of astrocytic gliomas of different malignancy grades Genes Chrom Cancer 22 915 9591629 10.1002/(sici)1098-2264(199805)22:1<9::aid-gcc2>3.0.co;2-1

[bib7] InoYSilverJSBlazejewskiLNishikawaRMatsutaniMvon DeimlingALouisDN1999 Common regions of deletion on chromosome 22q12.3-q13.1 and 22q13.2 in human astrocytomas appear related to malignancy grade J Neuropathol Exp Neurol 58 881885 10446812 10.1097/00005072-199908000-00010

[bib8] KleihuesPDavisRLOhgakiHBurgerPCWestphalMMCaveneeWK2000InPathology and Genetics of Tumours of the Nervous SystemKleihues P, Cavenee WK (eds)pp2239Lyon: IARC Press

[bib9] KonHSonodaYKumabeTYoshimotoTSekiyaTMurakamiY1998 Structural and functional evidence for the presence of tumor suppressor genes on the short arm of chromosome 10 in human gliomas Oncogene 16 257263 9464544 10.1038/sj.onc.1201488

[bib10] KarlbomAEJamesCDBoethiusJCaveneeWKCollinsVPNordenskjoldMLarssonC1993 Loss of heterozygosity in malignant gliomas involves at least three distinct regions on chromosome 10 Hum Genet 92 169174 8370584 10.1007/BF00219686

[bib11] LeungSYChanTLChungLPChanASFanYWHungKNKwongWKHoJWYuenST1998 Microsatellite instability and mutation of DNA mismatch repair genes in gliomas Am J Pathol 153 11811188 9777949 10.1016/S0002-9440(10)65662-3 PMC1853047

[bib12] LiJYenCLiawDPodsypaninaKBoseSWangSIPucJMiliaresisCRodgersLMcCombieRBignerSHGiovanellaBCIttmannMTyckoBHibshooshHWiglerMHParsonsR1997 PTEN, a putative protein tyrosine phosphatase gene mutated in human brain, breast, and prostate cancer Science 275 19431947 9072974 10.1126/science.275.5308.1943

[bib13] LibermannTANusbaumHRRazonNKrisRLaxISoreqHWhittleNWaterfieldMDUllrichASchlessingerJ1985 Amplification, enhanced expression and possible rearrangement of EGF receptor gene in primary human brain tumours of glial origin Nature 313 144147 2981413 10.1038/313144a0

[bib14] LipkinSMWangVStolerDLAndersonGRKirschIHadleyDLynchHTCollinsFS2001 Germline and somatic mutation analyses in the DNA mismatch repair gene MLH3: Evidence for somatic mutation in colorectal cancers Hum Mutat 17 389396 11317354 10.1002/humu.1114

[bib15] MiyakawaAIchimuraKSchmidtEEVarmeh-ZiaieSCollinsVP2000 Multiple deleted regions on the long arm of chromosome 6 in astrocytic tumours Br J Cancer 82 543549 10682663 10.1054/bjoc.1999.0961 PMC2363324

[bib16] MollenhauerJWiemannSScheurlenWKornBHayashiYWilgenbusKKvon DeimlingAPoustkaA1997 DMBT1, a new member of the SRCR superfamily, on chromosome 10q25.3-26.1 is deleted in malignant brain tumours Nat Genet 17 3239 9288095 10.1038/ng0997-32

[bib17] NishizakiTOzakiSHaradaKItoHAraiHBeppuTSasakiK1998 Investigation of genetic alterations associated with the grade of astrocytic tumor by comparative genomic hybridization Genes Chrom Cancer 21 340346 9559346 10.1002/(sici)1098-2264(199804)21:4<340::aid-gcc8>3.0.co;2-z

[bib18] O'ConnellPFischbachKHilsenbeckSMohsinSKFuquaSAClarkGMOsborneCKAllredDC1999 Loss of heterozygosity at D14S62 and metastatic potential of breast cancer J Natl Cancer Inst 91 13911397 10451444 10.1093/jnci/91.16.1391

[bib19] PershouseMAStubblefieldEHadiAKillaryAMYungWKSteckPA1993 Analysis of the functional role of chromosome 10 loss in human glioblastomas Cancer Res 53 50435050 8104691

[bib20] RasheedBKMcLendonREFriedmanHSFriedmanAHFuchsHEBignerDDBignerSH1995 Chromosome 10 deletion mapping in human gliomas: a common deletion region in 10q25 Oncogene 10 22432246 7784070

[bib21] ReifenbergerGLiuLIchimuraKSchmidtEECollinsVP1993 Amplification and overexpression of the MDM2 gene in a subset of human malignant gliomas without p53 mutations Cancer Res 53 27362739 8504413

[bib22] Reyes-MugicaMRieger-ChristKOhgakiHEkstrandBCHelieMKleinmanGYahandaAFearonERKleihuesPRealeMA1997 Loss of DCC expression and glioma progression Cancer Res 57 382386 9012460

[bib23] RosenbergJELisleDKBurwickJAUekiKvon DeimlingAMohrenweiserHWLouisDN1996 Refined deletion mapping of the chromosome 19q glioma tumor suppressor gene to the D19S412-STD interval Oncogene 13 24832485 8957092

[bib24] SallinenSLSallinenPHaapasaloHKononenJKarhuRHelenPIsolaJ1997 Accumulation of genetic changes is associated with poor prognosis in grade II astrocytomas Am J Pathol 151 17991807 9403731 PMC1858343

[bib25] SchmidtEEIchimuraKReifenbergerGCollinsVP1994 CDKN2 (p16/MTS1) gene deletion or CDK4 amplification occurs in the majority of glioblastomas Cancer Res 54 63216324 7987821

[bib26] SchrockEBlumeCMeffertMCdu ManoirSBerschWKiesslingMLozanowaTThielGWitkowskiRRiedTCremerT1996 Recurrent gain of chromosome arm 7q in low-grade astrocytic tumors studied by comparative genomic hybridization Genes Chrom Cancer 15 199205 8703845 10.1002/(SICI)1098-2264(199604)15:4<199::AID-GCC1>3.0.CO;2-X

[bib27] SmithJSTachibanaILeeHKQianJPohlUMohrenweiserHWBorellTJHosekSMSoderbergCLvon DeimlingAPerryAScheithauerBWLouisDNJenkinsRB2000a Mapping of the chromosome 19 q-arm glioma tumor suppressor gene using fluorescence in situ hybridization and novel microsatellite markers Genes Chrom Cancer 29 1625 10918389 10.1002/1098-2264(2000)9999:9999<::aid-gcc1007>3.3.co;2-9

[bib28] SmithJSTachibanaIPohlULeeHKThanarajasingamUPortierBPUekiKRamaswamySBillingsSJMohrenweiserHWLouisDNJenkinsRB2000b A transcript map of the chromosome 19q-arm glioma tumor suppressor region Genomics 64 4450 10708517 10.1006/geno.1999.6101

[bib29] SonodaYOzawaTHiroseYAldapeKDMcMahonMBergerMSPieperRO2001a Formation of intracranial tumors by genetically modified human astrocytes defines four pathways critical in the development of human anaplastic astrocytoma Cancer Res 61 49564960 11431323

[bib30] SonodaYOzawaTAldapeKDDeenDFBergerMSPieperRO2001b Akt pathway activation converts anaplastic astrocytoma to glioblastoma multiforme in a human astrocyte model of glioma Cancer Res 61 66746678 11559533

[bib31] SteckPAPershouseMAJasserSAYungWKLinHLigonAHLangfordLABaumgardMLHattierTDavisTFryeCHuRSwedlundBTengDHTavtigianSV1997 Identification of a candidate tumour suppressor gene, MMAC1, at chromosome 10q23.3 that is mutated in multiple advanced cancers Nat Genet 15 356362 9090379 10.1038/ng0497-356

[bib32] SuzukiTYokotaJMugishimaHOkabeIOokuniMSugimuraTTeradaM1989 Frequent loss of heterozygosity on chromosome 14q in neuroblastoma Cancer Res 49 10951098 2563671

[bib33] TongCYZhengPPPangJCPoonWSChangARNgHK2001 Identification of novel regions of allelic loss in ependymomas by high-resolution allelotyping with 384 microsatellite markers J Neurosurg 95 914 11453403 10.3171/jns.2001.95.1.0009

[bib34] TseJYNgHKLauKMLoKWPoonWSHuangDP1997 Loss of heterozygosity of chromosome 14q in low- and high-grade meningiomas Hum Pathol 28 779785 9224744 10.1016/s0046-8177(97)90149-0

[bib35] TsuzukiTTsunodaSSakakiTKonishiNHiasaYNakamuraM1996 Alterations of retinoblastoma, p53, p16(CDKN2), and p15 genes in human astrocytomas Cancer 78 287293 8674005 10.1002/(SICI)1097-0142(19960715)78:2<287::AID-CNCR15>3.0.CO;2-S

[bib36] UekiKOnoYHensonJWEfirdJTvon DeimlingALouisDN1996 CDKN2/p16 or RB alterations occur in the majority of glioblastomas and are inversely correlated Cancer Res 56 150153 8548755

[bib37] VoestenAMBijleveldEHWesterveldAHulsebosTJ1997 Fine mapping of a region of common deletion on chromosome arm 10p in human glioma Genes Chrom Cancer 20 167172 9331567

[bib38] VogelsteinBFearonERKernSEHamiltonSRPreisingerACNakamuraYWhiteR1989 Allelotype of colorectal carcinomas Science 244 207211 2565047 10.1126/science.2565047

[bib39] von DeimlingAEiblRHOhgakiHLouisDNvon AmmonKPetersenIKleihuesPChungRYWiestlerODSeizingerBR1992a p53 mutations are associated with 17p allelic loss in grade II and grade III astrocytoma Cancer Res 52 29872990 1349850

[bib40] von DeimlingALouisDNvon AmmonKPetersenIWiestlerODSeizingerBR1992b Evidence for a tumor suppressor gene on chromosome 19q associated with human astrocytomas, oligodendrogliomas, and mixed gliomas Cancer Res 52 42774279 1353411

[bib41] von DeimlingAFimmersRSchmidtMCBenderBFassbenderFNagelJJahnkeRKaskelPDuerrEMKoopmannJMaintzDSteinbeckSWickWPlattenMMullerDJPrzkoraRWahaABlumckeBWellenreutherRMeyer-PuttlitzBSchmidtOMollenhauerJPoustkaAStanglAPLenartzDvon AmmonK2000 Comprehensive allelotype and genetic analysis of 466 human nervous system tumors J Neuropathol Exp Neurol 59 544558 10850867 10.1093/jnen/59.6.544

[bib42] WatanabeKSatoKBiernatWTachibanaOvon AmmonKOgataNYonekawaYKleihuesPOhgakiH1997 Incidence and timing of p53 mutations during astrocytoma progression in patients with multiple biopsies Clin Cancer Res 3 523530 9815715

